# Localised Delivery of Cisplatin from Chitosan-Coated Titania Nanotube Array Nanosystems Targeting Nasopharyngeal Carcinoma

**DOI:** 10.34172/apb.2023.011

**Published:** 2022-01-05

**Authors:** Wan Nuramiera Faznie Wan Eddis Effendy, Rabiatul Basria S. M. N. Mydin, Amirah Mohd Gazzali, Srimala Sreekantan

**Affiliations:** ^1^Department of Biomedical Science, Advanced Medical and Dental Institute, Universiti Sains Malaysia, 13200 Bertam, Kepala Batas, Pulau Pinang, Malaysia.; ^2^School of Pharmaceutical Sciences, Universiti Sains Malaysia, 11800, Minden, Pulau Pinang, Malaysia.; ^3^School of Materials and Mineral Resources Engineering, Universiti Sains Malaysia, Engineering Campus, 14300, Nibong Tebal, Pulau Pinang, Malaysia.

**Keywords:** Cisplatin, Localised chemotherapeutic treatment, Nasopharyngeal carcinoma cells, Smart drug delivery, Titania nanotube arrays

## Abstract

***Pupose:*** Cisplatin (CDDP), while amongst the recognised chemotherapeutic drugs currently available, is known to have limitations; the lack of a single treatment approach and non-specific targeted therapies. Therefore, the development of an innovative strategy that could achieve localised CDDP treatment is an urgent undertaking. Recent advances in titania nanotube arrays (TNAs) technology have demonstrated promising applications for localised chemotherapeutic drug treatment. The present work investigated the efficiency of a TNA nanosystem for the localised CDDP treatment of nasopharyngeal carcinoma (NPC).

***Methods:*** Two models of the TNA nanosystem were prepared: CDDP loaded onto the TNA nanosystem surface (CDDP-TNA) and the other consisted of chitosan-coated CDDP-TNA. CDDP release from these two nanosystems was comprehensively tested on the NPC cells NPC/HK-1 and C666-1. The NPC cytotoxicity profile of the two CDDP-TNA nanosystems was evaluated after incubation for 24, 48 and 72 hours. Intracellular damage profiles were studied using fluorescence microscopy analysis with Hoechst 33342, acridine orange and propidium iodide.

***Results:*** The half-maximal inhibitory concentrations (IC_50_) of CDDP at 24 hours were 0.50 mM for NPC/HK-1 and 0.05 mM for C666-1. CDDP in the CDDP-TNA and chitosan-coated CDDPTNA models presented a significant degree of NPC inhibition (*P*<0.05) after 24, 48 and 72 hours of exposure. The outcome revealed cellular damage and shrinkage of the cell membranes after 48 hours of exposure to CDDP-TNA.

***Conclusion:*** This *in vitro* work demonstrated the effectiveness of TNA nanosystems for the localised CDDP treatment of NPC cells. Further *in vivo* studies are needed to support the findings.

## Introduction

 The potential applications of titania nanotube arrays (TNAs) in the biomedical field are well acknowledged.^1-3^ TNA possesses various properties that could address biomedical needs, such as enhanced interactions between nanosurfaces and cells, drug entrapment and controlled release^2^ and hydrophilic nanosurfaces, which could prevent bacterial adhesion.^3^ Antimicrobial drugs were previously explored extensively for drug loading into TNA with the goal of reducing post-implant surgery, which resulted in the implant rejection.^4^ The successful development of anti-microbial-loaded TNA opens new opportunities for loading chemotherapeutic drugs onto TNA,^5^ which was previously considered a tedious process because these drugs, especially those based on platinum,^6^ are light sensitive and carcinogenic. Cisplatin (CDDP) is a well-known chemotherapeutic drug for multiple cancers, such as nasopharyngeal carcinoma (NPC), gynaecological cancers, breast cancer and prostate cancer.^7^ Therefore, the present study was conducted to investigate the properties of CDDP loaded into TNA (CDDP-TNA) against NPC. A nanosystem containing the biopolymer chitosan was also constructed to achieve controlled CDDP release and prolong its cancer-killing effect.

**Figure 1:**
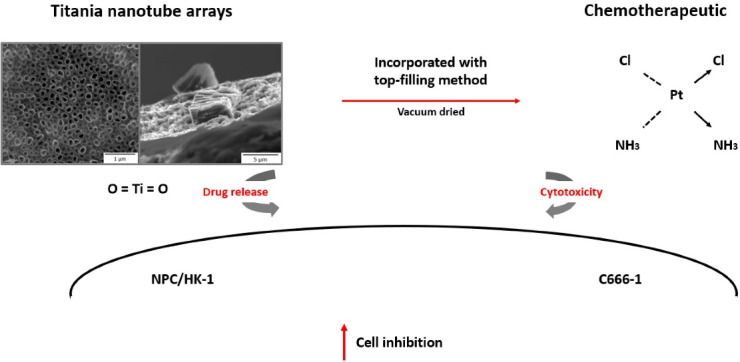
Illustration of the CDDP-TNA loading process and the cytotoxicity evaluation of NPC cells. CDDP was introduced to TNA surfaces through a top-filling method prior to *in vitro* assessment of its release profile and cytotoxicity evaluation

 NPC is a cancer that develops in the epithelial lining of the nasopharynx, a secluded location that has been known to be difficult to cure. In addition, drug resistance has also been documented in NPC cells, making treatment delivery more challenging^8^ thus in needed to an alternative for NPC treatment. The combined treatment of CDDP and some nano-materials, such as TNA, has been studied for localised cancer treatment. Researchers believe that the nano-scale size of TNA may be exploited to load and deliver CDDP to target cells. Chitosan coatings are natural polymers that feature several advantages,^9,10^ including strong anti-microbial property, good biocompatibility and easy degradability; the material is capable of controlled-drug release activity because of its positive charge, which promotes the opening of the mucosal passages and, thus, allows drug entry.^11^ Several factors have been reported to promote drug release from chitosan-coated TNAs, such as magnetic fields,^12^ temperature and pH^10^ and light,^13^ which degrades the biopolymer. Chitosan coatings have been reported to reduce the release rate of drugs loaded into TNAs^14^ so that drug release occurs over several hours or days.^15,16^ However, the intracellular damage caused by chitosan-coated CDDP-TNA, especially the types of damage resulting in cell death, has not yet been clearly described. This work describes the effect of chitosan-coated CDDP-TNA on NPC cells over time. The main objectives of this study are to evaluate the cytotoxicity of CDDP-TNA with and without a chitosan coating to NPC cells and screen the resulting cell damage by fluorescence microscopy analysis, as illustrated in Figure 1.

## Materials and Methods

###  Cell culture and study samples

 Epithelial cells of human NPC (NPC/HK-1) and (C666-1) were used in this work. NPC/HK-1 cell model, which originated from a recurrent NPC after radiotherapy, was given by Professor George Tsao from the University of Hong Kong. On the other hand, the C666-1 was an Epstein-Barr virus-related NPC cell was provided by Professor Kwok-Wai Lo from the Department of Anatomical & Cellular Pathology, Faculty of Medicine, The Chinese University of Hong Kong. The cells were maintained in supplemented RPMI 1640 media at 37ºC with 5% CO_2_ until they reached 80–90% confluency prior to cell culture work.

###  Adhesion analysis 

 The biocompatibility of the TNA nanosystem was analysed with a field emission scanning electron microscopy (FESEM) system (FEI Quanta FEG 650 with platinum (Pt) coating layer; Quorum-Q150T ES). A total of 1 × 10^5^ cells were seeded on top of TNA samples and filled with 500 µL culture media, then subsequently incubated at cell culture condition for 48 hours. Next, the culture media were removed and the cell-TNA was rinsed slowly with phosphate buffer saline (PBS) three times before being fixated with cold 2.5% (v/v) glutaraldehyde for 30 minutes. The fixative reagent was removed, and the cells-TNA were dehydrated with ethanol at increasing concentrations (50%, 70%, 90% and 99% [v/v]) for 10 minutes at each concentration. The samples were dried under airflow in a biosafety cabinet (BSC) and later stored at −20°C until visualisation through FESEM.

###  Cytotoxicity of the chitosan-coated CDDP-TNA nanosystem

 The anatase form of TNA was fabricated according to our previous work.^17^ Briefly, the titanium foil (Ti) undergoes electrochemical anodisation in an organic electrolyte and is loaded with CDDP. The concentration of CDDP to be loaded was determined by the amount of CDDP that caused 50% inhibition (IC_50_). CDDP diluted with culture media was pipetted out on top of TNA and vacuum dried for 90 minutes for each side at room temperature (RT). The final product is known as CDDP-TNA. Next, the preparation of chitosan was carried out following the method by Effendy et al.^18^ Chitosan-coated CDDP-TNA was obtained through the top filling method on both sides of CDDP-TNA and dried under airflow of BSC at RT for 2 hours, at least a day before the test.

 Following that, the cells were treated with different types of TNA nanosystem (TNA alone, CDDP-TNA and chitosan-coated CDDP-TNA) with untreated cells served as negative control and cells with CDDP alone as a positive control for 24, 48 and 72 hours. Cytotoxicity was evaluated by CellTiter 96 AQueous One Solution cell proliferation assay kit (Promega, USA), with absorbance measured by a microplate spectrophotometer reader (PowerWave^TM^ BioTek, USA) at 490 nm. Data analysis was conducted by using SPSS (version 26.0 for windows). Data were normalised to control (untreated cell) and expressed as mean ± standard deviation (SD), and at least two biological replicates were used. All data were statistically analysed using one-way ANOVA for IC_50_ determination and paired t-test to analyse *in vitro* cytotoxicity.

###  Intracellular activities of the TNA nanosystem

 Fluorescence microscopy was analysed using an Olympus Model 1X71 Fluorescence Microscope Phase Contrast and DP2-BSW (XV Image processing) software at 200 × 100 magnification. Hoechst 33 342, acridine orange (AO) and propidium iodide (PI) stains were used in observing cell damage upon the interaction of studied samples with cells.^10^ The cells were exposed to the various TNA nanosystem samples (refer to cytotoxicity part). The old media was removed, and cells were rinsed three times with PBS. After that, the samples were fixed with 70% [v/v] ethanol for 15 minutes. Ethanol was removed, and samples were exposed to Hoechst 33342 staining reagent for 30 minutes under the dark condition at RT. As for PI and AO, the PI reagent was first introduced and allowed to incubate with samples for 15 minutes. Next, the samples were rinsed with PBS, and AO staining was added and allowed to be incubated for another 15 minutes. The observation at different wavelengths was performed as a manufacturing protocol.

## Results and Discussion

###  Cell adhesion visualisation using FESEM

####  Cell adhesion visualisation by using FESEM

**Figure 2:**
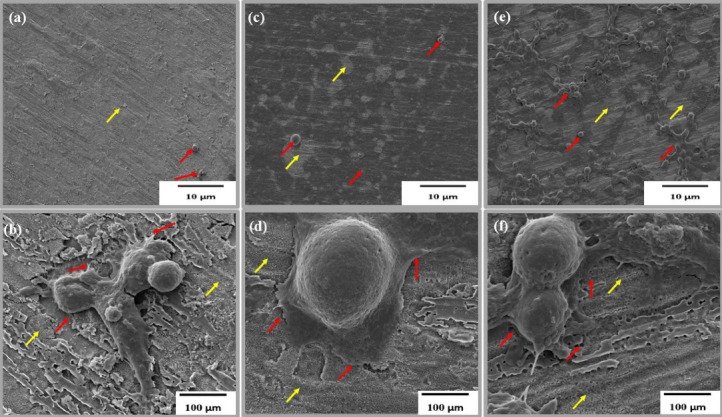
(a, c, e) Low-magnification wide-view and (b) high-magnification FESEM images of HEK 293 and NPC cells on TNA surfaces after 48 h of incubation. Yellow arrows indicate TNA, and red arrows indicate elongated filopodia in the cells. (a, b) HEK 293, (c, d) C666-1, (e, f) NPC/HK-1

 TNA nanosystems were successfully fabricated through anodisation at 30 V and 60 minutes, as described in detail in our previous work.^17,18^ Cell attachment to self-aligned TNA with an average inner diameter of 60 nm was successfully achieved, as shown in Figure 2; in the figure, the red arrow indicates elongated filopodia across the TNA, whilst yellow arrows indicate the nanotubes. C666-1 showed a higher degree of filopodium spreading compared with NPC/HK-1, as displayed in Figure 2c-f. Successful cell attachment and proliferation on the TNA surfaces suggests that the arrays have good biocompatibility and provide a favourable environment for cell growth, as reported in previous studies.^19,20^ In addition to NPC cells, HEK 293, a human embryonic kidney cell line, was also observed to confirm the biocompatibility of the TNA nanosystem with both normal and cancer cells (Figure 2a, b).

 Khaw et al^21^ reported that the diameter of the TNA is a key factor affecting cell adhesion and interaction. Previous reports summarised the influence of TNA diameter on cell proliferation, differentiation and gene expression in different type of cells. A diameter smaller than 30 nm seems to be more advantageous for cell interaction and proliferation activities than a larger diameter. However, Lü et al^22^ refuted this finding. Compared with 30 nm, TNA with 80 nm allow larger number of cells to grow. Li and Yang^23^ agreed that TNA with a diameter of 60 nm is effective in increasing cell adhesion and proliferation, consistent with our findings, as illustrated in Figure 2a-f. In most studies, cell attachment was usually investigated after 48 hours of incubation to enable the cells to adapt to their new environment.^24,25^ In general, the biocompatibility of the TNA nanosystem may be successfully observed through the presence of filopodia; this finding suggests a suitable environment for biomedical implants.

####  Cytotoxicity evaluation of NPC cells

**Figure 3:**
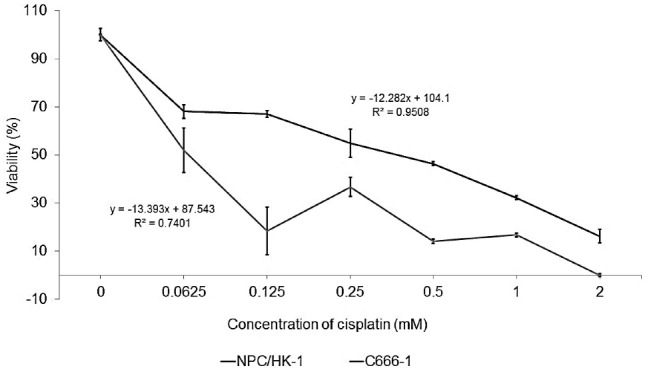
Cytotoxicity of CDDP towards the studied cells. IC_50_ values were calculated and determined from the graph. The IC_50_ values of CDDP for NPC/HK-1 and C666-1 were 0.50 and 0.05 mM, respectively. These values were then applied to the study on CDDP-TNA release

 The cytotoxicity of CDDP to NPC and HEK 293 cells was measured, as shown in Figure 3 (a, b). The IC_50_ values of CDDP for NPC/HK-1 and C666-1 were 0.50 and 0.05 mM, respectively. IC_50_ was calculated using the equation determined from graph (Figure 3a, b) respectively. The CDDP concentration was kept constant throughout the *in vitro* studies to enable the examination of the detailed effect of the working CDDP concentration on NPC cells.

 NPC/HK-1 appeared to be less sensitive to CDDP than C666-1, as reflected in Figure 3a, possibly because of its origin as a recurrent cancer.^26^ The C666-1, an EBV-related NPPC was more susceptible to the chemotherapeutic drug treatment, resulting in a lower IC_50_ of CDDP-treated cells. In comparison to C666-1, the resistance of NPC/HK-1 towards CDDP treatment was previously noted by Daker et al.^27^ In the same report, Daker also discussed on the higher sensitivity of C666-1 compared to NPC/HK-1 towards the NPC treatment given thus confirming the resistance of NPC/HK-1 towards cancer treatment. Earlier reports also discussed the importance of using other chemotherapeutic drugs, such as the combination of CDDP and 5-fluorouracil, to treat NPC/HK-1 effectively.^27,28^ However, according to Low et al,^29^ undifferentiated C666-1 appears to be less sensitive towards CDDP treatment compared with differentiated NPC/HK-1. This phenomenon could be explained by the involvement of *DUSP16* in the response of NPC towards CDDP treatment. Prolonged and greater exposure of undifferentiated C666-1 to CDDP causes an increase in the expression of DUSP16 mRNA, thereby resulting in the greater resistance of these cells towards CDDP compared with NPC/HK-1. This contradiction in outcomes suggests the need for more comprehensive studies on the effect of CDDP on NPC cells in future work.

###  Evaluation of the cytotoxicity of CDDP-TNA to NPC cells

**Figure 4:**
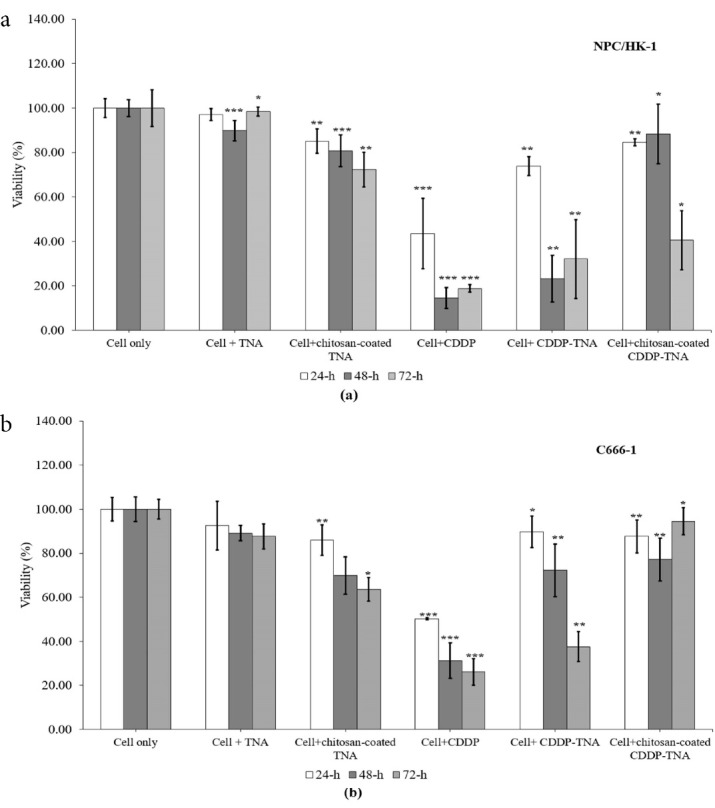
*In vitro* cytotoxicity of CDDP-TNA and chitosan-coated CDDP-TNA to (a) NPC/HK-1 and (b) C666-1. Significant inhibition was observed and noted with an asterisk

 The inhibition rates of chitosan-coated CDDP-TNA for NPC/HK-1 ranged from 84.60% ± 1.54% (24 hours) to 40.54% ± 13.2% (72 hours), thereby demonstrating the sustained-release effects of CDDP in the cell culture over time. The decrease in cell viability after exposure to CDPP-TNA was fairly prominent, with values of 73.88% ± 4.21% (24 hours), 23.22% ± 10.49% (48 hours) and 32.09% ± 17.74% (72 hours), as displayed in Figure 4a. This result suggests the ability of chitosan to prolong the release of CDDP and reducing the burst release of CDDP after the initial interaction. The inhibition of NPC/HK-1 afforded by CDDP-TNA, CDDP-TNA and chitosan-coated CDDP-TNA showed significant differences, and *P* values of < 0.05 were obtained in all periods studied. This finding illustrates the effectiveness of chitosan in achieving the sustained and continuous release of CDDP from the chitosan-coated CDDP-TNA nanosystem. Therefore, this system may have potential applications in future cancer treatment.

 The inhibition rates of CDDP-TNA for C666-1 were 89.76% ± 7.10% (24 hours), 72.24% ± 11.88% (48 hours) and 37.59% ± 6.80% (72 hours). Moreover, different patterns of inhibition were observed in cells exposed to chitosan-coated CDDP-TNA, the inhibition rates of which were 87.62% ± 7.40% (24 hours), 77.14% ± 9.80% (48 hours) and 94.52% ± 6.19% (72 hours). Significant inhibition of C666-1 (*P* < 0.05) was observed in all CDDP treatments regardless of the period of incubation, thereby suggesting the effectiveness of the drug in killing NPC cells (Figure 4b). Cells exposed to CDDP-TNA and chitosan-coated CDDP-TNA presented significant inhibition compared with untreated cells, thus confirming the effectiveness of the TNA nanosystem in delivering CDDP towards target sites over prolonged periods. The significant inhibition (*P* < 0.05) of cells treated with CDDP-TNA with and without the chitosan coating could be observed in the Figure 4 showed no significant difference in cell inhibition following exposure to the TNA nanosystem was noted. The inhibition of cells exposed to CDDP-TNA and chitosan-coated CDDP-TNA suggested that initial release of the CDDP from TNA had taken place, thereby proving the effectiveness of CDDP-TNA for CDDP loading, slow release and cell death.

 The inhibition pattern was observed in all cells significantly inhibited by CDDP (*P* < 0.05). TNA alone showed no significant inhibition (*P* > 0.05) of any of the cells in the first 24 hours, which supports the suitability of this nano-material as a CDDP delivery nanosystem. The free TNA nanosystem demonstrated inhibitory effects on NPC cells, in agreement with the findings of Mohamed et al.^30^ In the present work, self-aligned TNA was grown and fixed onto a Ti substrate, which may explain the low inhibitory effect of this material on cancer cells and supports the biocompatibility of TNA. Compared with CDDP alone, chitosan-coated CDDP-TNA prolonged and sustained CDDP release into the study environment, resulting in a small reduction in the number of cells. Other factors that could influence the slow release of drugs are related to the morphology of the TNA.^31^ Optimisation of the size of the TNA nanosystem for CDDP loading was performed (data not shown here) by employing a longer anodisation period to obtain longer nanotubes, thus promoting longer release activity, as reflected by the percentage of cell survival after 72 hours ( > 40%) compared with CDDP alone (approximately 20%).

###  Intracellular activities of cells against the TNA nanosystems

 The severity of cell damage was evaluated by monitoring changes in cell morphology, size and staining intensity. Cell survival after the addition of CDDP was assessed, and the cell membrane was observed. Bright-field analysis showed cell shrinkage and debris surrounding the cells^32^ after treatment with chitosan-coated TNA, CDDP, CDDP-TNA and chitosan-coated CDDP-TNA. The pale blue tone suggested a decrease in double-stranded DNA due to cell death, and AO/PI staining showed extensive colour distribution, which reflects changes in cell morphology.

**Figure 5:**
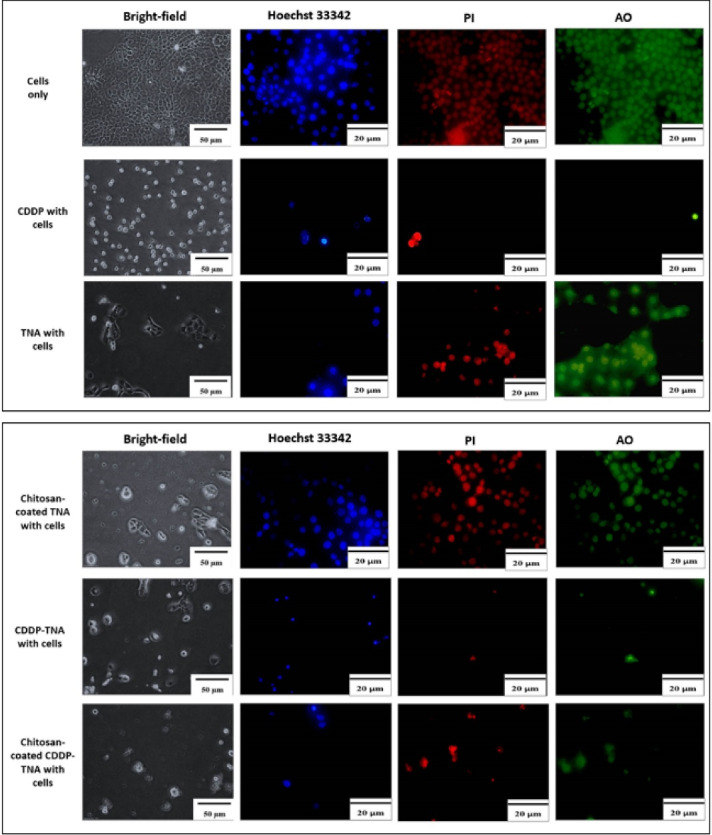
Microscopic analysis of NPC/HK-1. Bright-field microscopic analysis, Hoechst 33342, PI, and AO staining were applied to observe the aftereffect on cells upon interaction with the samples. The ability of the CDDP-TNA in maintaining the functionality of CDDP was demonstrated by cell morphological changes, high fluorescence intensity, and the presence of cell fragments indicating cell damage

**Figure 6:**
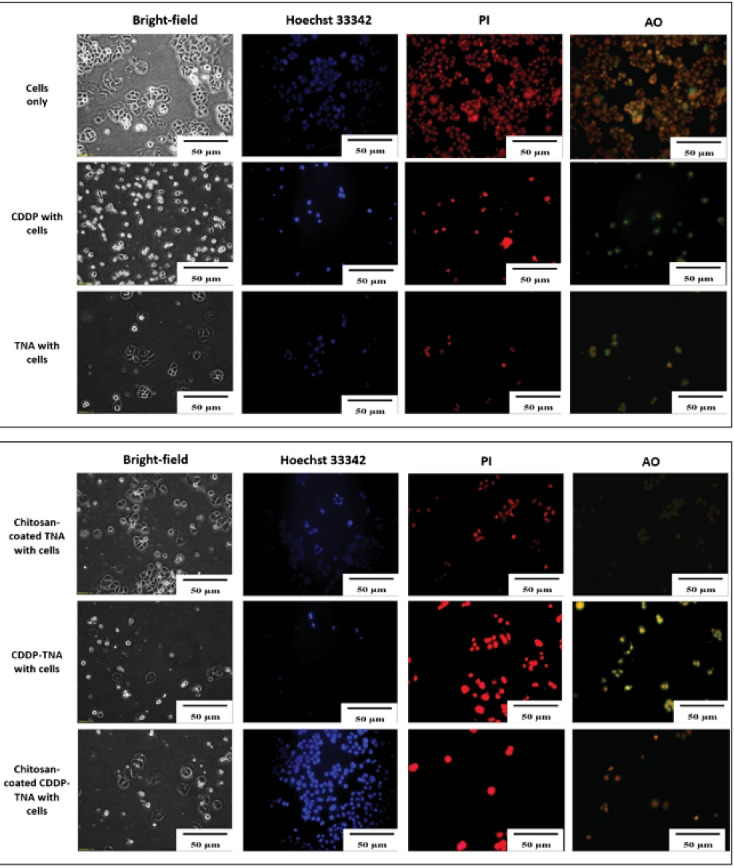
Intracellular activities of C666-11 cells against the TNA nanosystem. Cell damage upon treatment with CDDP-TNA in the absence or presence of chitosan could be observed *via *the expression of red and orange–green cells. Analysis with an inverted light microscope showed a small number of cells. This result indicates the destruction of cancer cells

 The presence of cell fragments of NPC cells treated with CDPP-TNA and chitosan-coated CDDP-TNA in Figures 5 and 6 indicates cell damage caused by CDDP-TNA. The findings corroborated the impact of CDDP, which induced cell damage, while the cells subjected to TNA and chitosan-coated TNA revealed no presence of cell fragments indicated no cell damage upon interaction which those two samples. These changes were not observed in cells treated with CDDP-TNA and chitosan-coated CDP-TNA, and different intensities of the bluish tone of the cells were noted. Fluorescent staining analysis was performed to confirm cell apoptosis, which observed through Hoechst 33 342 staining. The results obtained from NPC cells were similar to those obtained from cells treated with CDDP-TNA and chitosan-coated CDDP-TNA, thus confirming that cell apoptosis had occurred. These findings demonstrate the effectiveness of TNA for drug loading and its roles in promoting the release of CDDP and killing NPC cells.

 The presence of cell fragments helped achieve the successful qualification of staining outcomes displayed in the images of cells treated with CDDP-TNA and chitosan-coated CDDP-TNA. AO/PI was used as an indicator of cell damage to evaluate the mechanism of CDDP in killing cancer cells. In general, CDDP destroys double-stranded DNA,^33,34^ thereby effectively causing damage to cancer cells. The outcome of the intracellular activities of these cells showed that CDDP was successfully delivered from the TNA nanosystem and effectively killed cancer cells. On the other hand, presence of chitosan had successfully demonstrated slow kill of NPC cells up to 72 hours.

## Conclusion

 The cellular damage profiles obtained in this work demonstrate that chitosan-coated CDDP-TNA nanosystems feature sustained CDDP release properties and slow-killing effects on NPC cells. This research suggests that TNA nanosystems could be useful for localised chemotherapeutic drug treatment. Further research on the molecular characteristics of NPC cells exposed to chitosan-coated CDDP-TNA will enhance the current understanding of the mechanism of cell death. More *in vivo* studies could also promote the effectiveness of drugs developed for cancer treatment.

## Acknowledgments

 The authors would like to thank Universiti Sains Malaysia (RUI EKSESAIS 2019 (No:1001/CIPPT/8012338) for sponsoring this work.

## Author Contributions

**Conceptualisation**: Rabiatul Basria S. M. N. Mydin.

**Datacuration**: Rabiatul Basria S. M. N. Mydin, Wan Nuramiera Faznie Wan Eddis Effendy.

**Formalanalysis**: Wan Nuramiera Faznie Wan Eddis Effendy, Rabiatul Basria S. M. N. Mydin, Amirah Mohd Gazzali.

**Fundingacquisition**: Rabiatul Basria S. M. N. Mydin.

**Investigation**: Wan Nuramiera Faznie Wan Eddis Effendy, Rabiatul Basria S. M. N. Mydin.

**Methodology**: Wan Nuramiera Faznie Wan Eddis Effendy, Rabiatul Basria S. M. N. Mydin, Amirah Mohd Gazzali, Srimala Sreekantan.

**ProjectAdministration**: Wan Nuramiera Faznie Wan Eddis Effendy, Rabiatul Basria S. M. N. Mydin, Amirah Mohd Gazzali, Srimala Sreekantan.

**Resources**: Rabiatul Basria S. M. N. Mydin, Amirah Mohd Gazzali, Srimala Sreekantan.

**Software**: Wan Nuramiera Faznie Wan Eddis Effendy, Rabiatul Basria S. M. N. Mydin, Srimala Sreekantan.

**Supervision**: Rabiatul Basria S. M. N. Mydin, Amirah Mohd Gazzali, Srimala Sreekantan.

**Validation**: Wan Nuramiera Faznie Wan Eddis Effendy, Rabiatul Basria S. M. N. Mydin, Amirah Mohd Gazzali, Srimala Sreekantan.

**Visualisation**: Wan Nuramiera Faznie Wan Eddis Effendy, Rabiatul Basria S. M. N. Mydin, Amirah Mohd Gazzali, Srimala Sreekantan.

**Writing – originaldraft**: Wan Nuramiera Faznie Wan Eddis Effendy, Rabiatul Basria S. M. N. Mydin, Amirah Mohd Gazzali, Srimala Sreekantan.

**Writing – review & editing**: Wan Nuramiera Faznie Wan Eddis Effendy, Rabiatul Basria S. M. N. Mydin.

## Ethical Issues

 The authors declared no ethical issues related to this works.

## Conflict of Interest

 All authors declared no conflict of interest regarding the sources and funding.
